# Draft Genome Sequences of 29 Helicobacter pylori Strains Isolated from Colombia

**DOI:** 10.1128/MRA.00218-21

**Published:** 2021-05-13

**Authors:** Angela B. Muñoz, Johanna Stepanian, Carmen Acosta, Juan S. Solano-Gutierrez, Filipa F. Vale, Alba A. Trespalacios-Rangel

**Affiliations:** a Infectious Diseases Research Group, Microbiology Department, Sciences Faculty, Pontificia Universidad Javeriana, Bogotá, Colombia; b AXOMICS-Sequencing Center, Biological Science Department, Science School, Universidad EAFIT, Medellín, Colombia; c Host-Pathogen Interactions Unit, Research Institute for Medicines (iMed-ULisboa), Faculdade de Farmácia, Universidade de Lisboa, Lisbon, Portugal; Loyola University Chicago

## Abstract

Here, we present the draft genome sequences of 29 Colombian Helicobacter pylori strains. These strains were isolated in Bogotá, Colombia, from patients diagnosed with chronic gastritis. The genomic characterization of these strains will provide more information on the genetic composition of H. pylori strains from Colombia.

## ANNOUNCEMENT

Helicobacter pylori is a Gram-negative, pathogenic bacterium capable of colonizing and persisting in the human stomach. The infection is considered the most frequent chronic bacterial infection worldwide (1–3), reaching prevalence rates of up to 80% in Colombia (4).

This report announces the genome sequences of 29 H. pylori strains isolated between 2009 and 2010 from patients residing in Bogotá, Colombia. The patients who signed informed consent were 48 years old on average (range, 18 to 79 years); from the histology results, 65.5% were diagnosed with chronic nonatrophic gastritis and 34.5% with chronic atrophic gastritis. The strains were recovered from gastric biopsy samples, and those were cultivated on BBL Brucella agar (Becton, Dickinson) supplemented with 7% horse blood, 0.4% IsoVitalex (BD, USA), and 0.2% Dent selective supplement (Oxoid, UK) under microaerophilic conditions (11% CO_2_) at 37°C for 4 to 7 days. The strains were preserved in 20% glycerol and stored until required for DNA extraction. They were recovered by culture every time. After that, total DNA was extracted using a DNeasy blood and tissue kit (Qiagen, Hilden, Germany) following the manufacturer’s instructions. Fluorometric assay DNA quantification was performed using a Qubit 2.0 fluorometer and the Qubit double-stranded DNA (dsDNA) high-sensitivity (HS) assay kit (Life Technologies, Carlsbad, CA, USA). To verify that the DNA obtained was from H. pylori, a conventional PCR technique for the *vacA* gene was carried out. The primers and protocols previously described by Atherton et al. (5) were used.

Genomic DNA was sequenced using the MiSeq platform (Illumina, San Diego, CA); DNA libraries were prepared using a Nextera XT DNA library preparation kit (Illumina), followed by 2 × 300-bp paired-end sequencing resulting in 80× coverage. The low-quality sequences were removed with the software package Trimmomatic v0.39 (6). The reads were used for *de novo* genome assembly with SPAdes v13.3 (7). Assembly statistics for analyzed strains are provided in Table 1. The sequences were annotated using the NCBI Prokaryotic Genome Automatic Annotation Pipeline (PGAAP) (8). Default parameters were used for all software tools unless otherwise specified.

**TABLE 1:** Genome statistics of sequences reported

Strain name	GenBank accession no.	SRA accession no.	No. of CDS^*a*^	Genome size (bp)	GC content (%)	MLST	No. of contigs	*N*_50_ value (bp)	Genome coverage (×)	No. of raw reads
COL 1-PUJ	JAFCHS000000000	SRR13796410	1,582	1,679,429	38.8	HpEurope	72	58,624	135	1,163,956
COL 2-PUJ	JACSDV000000000	SRR13796434	1,632	1,607,581	39	HpEurope	46	82,542	392	2,633,600
COL 5-PUJ	JACSDU000000000	SRR13796433	1,667	1,624,361	39.1	HpEurope	133	21,560	105	850,310
COL 6-PUJ	JACSDT000000000	SRR13796422	1,673	1,625,175	39.1	HpEurope	133	20,478	95	745,268
COL 8-PUJ	JAFCHT000000000	SRR13796409	1,664	1,661,424	38.9	HpEurope	43	93,756	286	2,823,146
COL 9-PUJ	JAFCHU000000000	SRR13796408	1,653	1,613,788	39	HpEurope	47	84,065	311	2,073,864
COL 10-PUJ	JAFCHV000000000	SRR13796407	1,680	1,642,843	39.4	HpEurope	58	107,835	375	2,741,078
COL 11-PUJ	JAFCHW000000000	SRR13796406	1,663	1,626,191	39.4	HpEurope	58	86,547	221	1,639,572
COL 12-PUJ	JAFCHX000000000	SRR13796432	1,633	1,637,127	38.9	HpEurope	43	96,559	368	2,773,188
COL 13-PUJ	JAFCHY000000000	SRR13796431	1,714	1,658,899	39.3	HpEurope	58	59,276	554	4,615,312
COL 14-PUJ	JAFCHZ000000000	SRR13796430	1,701	1,673,807	38.9	HpEurope	54	80,670	256	1,758,244
COL 15-PUJ	JAFCIA000000000	SRR13796429	1,563	1,546,556	39.2	HpEurope	46	60,019	133	987,226
COL 16-PUJ	JACSDS000000000	SRR13796412	1,665	1,634,541	39.3	HpEurope	64	54,632	482	2,363,616
COL 18-PUJ	JAFCIB000000000	SRR13796428	1,662	1,658,604	38.9	HpEurope	37	93,756	580	436,440
COL 19-PUJ	JAFCIC000000000	SRR13796427	1,654	1,656,342	38.9	HpEurope	42	81,649	371	2,363,966
COL 20-PUJ	JAFCID000000000	SRR13796426	1,674	1,619,537	39	HpEurope	56	59,593	340	2,201,658
COL 21-PUJ	JAFCIE000000000	SRR13796425	1,670	1,618,229	39	HpEurope	55	58,359	435	3,168,290
COL 23-PUJ	JACSDR000000000	SRR13796411	1,661	1,614,131	39.1	HpEurope	91	32,948	99	762,738
COL 24-PUJ	JAFCIF000000000	SRR13796424	1,674	1,622,244	39	HpEurope	119	25,019	119	922,348
COL 25-PUJ	JAFCIG000000000	SRR13796423	1,681	1,662,282	38.9	HpEurope	88	37,899	113	909,004
COL 26-PUJ	JAFCIH000000000	SRR13796421	1,631	1,619,895	39	HpEurope	52	97,025	208	1,447,706
COL 27-PUJ	JAFCII000000000	SRR13796420	1,656	1,653,551	38.9	HpEurope	36	80,500	359	2,247,256
COL 28-PUJ	JAFCIJ000000000	SRR13796419	1,671	1,653,809	38.9	HpEurope	23	132,947	479	3,095,502
COL 29-PUJ	JAFCIK000000000	SRR13796418	1,585	1,586,826	39.2	HpEurope	31	144,624	340	2,443,034
COL 30-PUJ	JAFCIL000000000	SRR13796417	1,596	1,587,263	39.1	HpEurope	35	92,323	339	2,478,576
COL 31-PUJ	JAFCIM000000000	SRR13796416	1,795	1,711,739	39	HpEurope	14	41,620	363	2,430,084
COL 49-PUJ	JAFCIN000000000	SRR13796415	1,786	1,735,252	39	HpEurope	96	59,657	255	2,107,660
COL 50-PUJ	JAFCIO000000000	SRR13796414	1,726	1,711,312	38.9	HpEurope	57	118,609	522	4,410,236
COL 51-PUJ	JAFCIP000000000	SRR13796413	1,656	1,666,802	38.9	HpEurope	39	82,225	404	2,801,844

a CDS, coding DNA sequences.

A multilocus sequence typing (MLST) analysis was performed based on seven H. pylori housekeeping genes (*atpA*, *efp*, *trpC*, *ppa*, *mutY*, *yphC*, and *urel*). The sequences of these genes from 741 strains available at PubMLST (http://pubmlst.org/helicobacter/) (9) and previously described by Falush et al. (10) and Linz et al. (11), plus the 29 strains included in this study, were aligned using MAFFT v7 (12). Then, the aligned sequences were analyzed in the Structure 2.3.4 software (13–15) and the MEGA 7.0 software (16). For these analyses, previously reported recommendations (17, 18) were followed, and the results revealed that all Colombian isolates included in this study were classified as HpEurope.

The data reported here provide information on the genetic population structure of Colombian H. pylori. This information will help future functional comparative genomic studies that will greatly enhance the understanding of H. pylori infection dynamics in the Latin American region.

### Data availability.

This whole-genome shotgun project has been deposited in GenBank under accession number PRJNA656306. The accession numbers for the genomes are provided in Table 1.
